# What do patients do during a familial Mediterranean fever attack? Their strategies and associated factors

**DOI:** 10.1007/s11739-025-04039-6

**Published:** 2025-07-01

**Authors:** Özlem Kılıç, Seda Çolak, Emre Tekgöz, Mehmet Nur Kaya, Muhammet Çınar, Sedat Yılmaz

**Affiliations:** 1 Department of Rheumatology, Ankara Training and Research HospitalAnkara Training and Research Hospital, University of Health Sciences, Hacettepe, Ulucanlar Street, No:89, 06230 Altındağ/Ankara, Türkiye; 2 Department of Internal Medicine, Division of Rheumatology, Gülhane Training and Research Hospital, University of Health Sciences , Ankara, Türkiye

**Keywords:** Familial Mediterranean fever, Attack, Mitigating strategies, Emergency department, Colchicine

## Abstract

Familial Mediterranean fever (FMF) is an autosomal recessive autoinflammatory disease characterised by recurrent fever and serositis. Despite colchicine’s proven efficacy, attacks may persist, necessitating the implementation of various mitigation strategies by patients.  To identify attack mitigation strategies and associated factors in FMF patients. A cross-sectional study at a tertiary rheumatology clinic included adults (≥ 18 years) meeting Tel-Hashomer criteria, on colchicine for ≥ 6 months, with ≥ 1 attack in the past six months. Patients were asked about their attack mitigation strategies, including colchicine dose increase, emergency department (ED) admission, resting, abdominal hot pack, dietary changes, on-demand anakinra, herbs, paracetamol, non-steroidal anti-inflammatory drugs (NSAIDs), and corticosteroid use. Among 258 patients (98 men, 160 women; median age 35 years, disease duration 18 years), 93% used mitigation strategies. The most common first choice was ED admission (19.8%), followed by colchicine dose increase (18.2%), resting (14.3%), dietary changes (4.3%), NSAIDs (7%), paracetamol (6.6%), on-demand anakinra (6.2%), herbs (6.2%), and corticosteroids (5%). Normal C-reactive protein (CRP) levels, shorter last attack duration, lower international Severity Score for FMF (ISSF), non-working and non-M694V mutations were linked with colchicine dose increase (p < 0.05). M694V positivity, working, persistent CRP elevation, dominant serositis and musculoskeletal attacks, education level ≤ 8 years, longer last attack duration, higher ISSF, current colchicine resistance and non-adherence were linked with ED admission (p < 0.05). Most FMF patients used strategies to mitigate attacks, mainly ED admission and colchicine dose increase. These were influenced by genetic mutations, attack type, working status, education, and CRP levels.

**Supplementary Information:**

The online version contains supplementary material available at 10.1007/s11739-025-04039-6.

## Introduction

Familial Mediterranean Fever (FMF) is an inherited autoinflammatory disease that predominantly affects people of Mediterranean origin, such as Turks, Arabs, Armenians and Jews [1, 2]. It is characterised by recurrent episodes of fever and serositis, which mainly affect the peritoneum, the pleura and the joints [3]. Attacks typically last between 24 and 72 h [4]. The disease is caused by mutations in the Mediterranean FeVer (MEFV) gene, which encodes the pyrin protein. This leads to overactivation of the pyrin inflammasome and overproduction of interleukin-1β, resulting in inflammation [3, 4]. Persistent inflammation can lead to complications such as renal amyloidosis and end-stage renal disease [4].

Colchicine is the cornerstone of pharmacological treatment, while lifestyle changes and monitoring play an important role in non-pharmacological management. However, some patients continue to experience attacks despite receiving colchicine treatment. This may be due to drug resistance, side effects of the treatment that limit the optimal dosage, poor adherence to the treatment plan, or genetic factors [5]. Identifying these causes is important for improving patient outcomes and managing the disease. However, it is also likely that various methods will be employed to alleviate attacks occurring during treatment. This is because even a single attack can have a negative impact on a patient’s quality of life and work performance [6]. During a FMF attack, the focus of acute care is largely on symptom management and support. This usually involves providing intravenous fluids, antipyretics and NSAIDs [7].

Increasing colchicine doses during FMF attacks is a common practice, especially in patients who do not respond adequately to standard doses. Increasing the dose of colchicine can lead to a significant reduction in the severity of attacks. However, the decision to increase the dose should be carefully considered due to potential side effects and individual patient [8]. If patients are resistant to colchicine, IL-1 inhibitors such as anakinra and canakinumab are used [3, 4]. In some cases, alternative therapies such as tumor necrosis factor inhibitors for chronic arthritis and IL-6 inhibitors for amyloidosis may be used [3, 4]. Opinions on the use of IL-1 antagonists in the treatment of FMF may differ depending on the clinical picture of the patient; some advocate continuous use, while others recommend on-demand use [2, 9]. Although corticosteroids are not a first-line treatment but can be used in the acute setting to treat severe symptoms [10, 11]. Similarly paracetamol is not a first-line treatment for FMF, it can be used for relief of symptoms such as fever and pain during acute attacks [11, 12]. Non-steroidal anti-inflammatory drugs (NSAIDs) are commonly used in the treatment of FMF to provide symptomatic relief of pain and inflammation, especially during acute attacks [11–13]. FMF is an autoinflammatory disease that frequently leads to emergency department (ED) admissions for acute abdominal pain and other symptoms. The decision to present to the ED during an attack of FMF is influenced by the severity of symptoms, potential complications and the need for differential diagnosis to rule out other conditions [14]. ED often uses intravenous/intramuscular NSAIDs and intravenous fluids to manage acute symptoms [15]. Although there are no specific dietary recommendations for the management of FMF attacks, it is generally recommended that a balanced diet is maintained to support overall health and possibly reduce the severity of the attack [16]. There are no specific herbal recommendations for managing FMF attacks, however several case reports have highlighted the use of herbal therapies in FMF management [17, 18]. FMF attacks often mimic acute abdominal discomfort with severe pain and stiffness [13]. The use of abdominal hot packs has not been explicitly discussed in the literature, but it can be seen to be used as a potential complementary therapy for symptom relief during abdominal attacks of FMF. The importance of rest in the treatment of FMF attacks has been highlighted [19].

To our knowledge, no study has been published in the literature investigating the methods used by patients to reduce the severity of an attack. Therefore, the aim of this study was to survey a group of FMF patients to elucidate their most frequently preferred strategies for mitigating their attacks and to investigate the clinical, demographic, laboratory and genetic characteristics associated with the reported strategies.

## Methods

### Participants and study design

The cross-sectional study was conducted in a tertiary rheumatology outpatient clinic. Patients aged 18 years and older who were diagnosed with FMF according to Tel-Hashomer criteria between August 2023 and February 2024 and who had received colchicine treatment for at least six months were evaluated. Those who signed the written informed consent form and had experienced at least one attack in the previous six months were included in the study. Patients who did not attend regular follow-up visits at least twice a year with an interval of at least 6 months, patients who used colchicine for less than 6 months, patients with missing data, patients who developed side effects that limited the continued use of colchicine, patients under 18 years of age, and pregnant patients were excluded from the study.

### Calculation of sample size

A power analysis was conducted using the G*Power software (version 3.1.9.6, developed by Franz Faul at the University of Kiel, Germany). The primary outcome was the prevalence of attack-mitigating strategies among FMF patients, which was estimated at 82%. With an effect size of 0.1, an alpha error of 0.05, and a statistical power of 0.95, the required sample size was calculated to be at least 227 individuals. Ultimately, the study was completed with 258 FMF patients.

### Laboratory and clinical assessment

Patients were assessed and examined by a 3-year-experienced rheumatologist. During a face-to-face visit in a quiet room, participants were asked to answer questions posed by the researcher. The assessment of each participant took about 30 min. Data on clinical, treatment, laboratory, and demographic characteristics were collected from the patients and their medical records at the time of admission. Clinical and demographic characteristics included age, sex, age at onset/diagnosis, disease duration, diagnosis duration, delay in diagnosis (time from symptom onset to diagnosis), working status, education level (8 years and below, 9 to 12 years, 12 years and above), attack characteristics (fever, erysipelas-like erythema, pleuritis, febrile myalgia, peritonitis, pericarditis, diarrhoea, and arthritis), dominant attack types (serositis and musculoskeletal), family history (parental consanguinity and family history of FMF), current smoking status, history of appendectomy, colchicine dose (maximum tolerated colchicine dose in non-responders), colchicine treatment adherence, response to colchicine, current colchicine resistance, use of biologic therapy due to a history of colchicine resistance, International Severity Score for FMF (ISSF) and duration of last attack. The diagnosis and disease duration were calculated by subtracting the age at diagnosis and symptom onset from the patient’s age. MEFV mutation results were obtained from medical records. The persistent elevated CRP was characterized by CRP levels ≥ 5 mg/L in more than 80% of patient visits lasting at least one year [20]. CRP levels below 5 mg/L at these visits were noted as normal CRP level. The majority of visits involving persistently elevated CRP occurred within the last 3 months. Patients were evaluated etiologically during follow-up. Identifiable etiological causes included frequent attacks due to colchicine resistance and treatment inadherence. At the end of the assessment, these patients received education on treatment adherence and side effect management, as well as an anti-IL-1 treatment plan if necessary.

Chest radiography and echocardiography results were available for patients with a history of pleurisy or pericarditis. These findings were recorded in the patients’ medical records by their attending rheumatologist. These data were obtained from existing medical records. Current colchicine resistance was defined as one or more attacks per month for the previous 3 months, despite treatment with the maximum tolerated dose of colchicine [21].

### Disease severity

The ISSF was used for the assessment of disease severity. It was developed for clinical practice and research purposes to measure disease severity in adult and pediatric FMF patients. It consists of nine variables including nine laboratory and clinical parameters: organ dysfunction, organ failure, attack frequency, chronic sequelae, involvement of more than two sites during a single episode, increased acute phase reactants, more than two different types of attacks during the course of the disease, exertional leg pain and duration of attacks. The maximum score is 10 and severity is defined as mild (≤ 2), moderate (3–5) or severe disease (≥ 6) [22].

### Assessment of colchicine adherence

Medication adherence was assessed using the Modified Morisky Scale (MMS). It is a short, simple and reliable test with Turkish validity and reliability. It consists of 6 questions. Answers were scored as ‘yes’ or ‘no’; in the 2nd and 5th questions, a ‘yes’ answer was scored as 1 point and a ‘no’ answer was scored as 0 point; in the other questions, a ‘yes’ answer was scored as 0 point and a ‘no’ answer was scored as 1 point. A total score of 0 or 1 in questions 1, 2 and 6 indicates a low level of motivation, while scores above 1 point indicate a high level of motivation. A total score of 0 or 1 for questions 3, 4 and 5 indicates a low level of knowledge, and scores above 1 point indicate a high level of knowledge. Low motivation and low knowledge indicate non-adherence. High motivation and knowledge indicate adherence [23].

### Strategies that mitigated attacks

Patients with FMF who had been receiving colchicine treatment for at least six months were asked to indicate which strategy they had used first to alleviate their attacks in the previous six months. Long-term follow-up is recommended in order to assess the success of, and adherence to, colchicine treatment for FMF [24]. The EULAR treatment recommendations specify this period as six months [25]. Considering the criterion of at least 6 months of colchicine treatment and a 6 month study period, all patients had been receiving colchicine treatment for at least 1 year. The strategies that patients used to mitigate their attacks were listed in a questionnaire under the following headings: Colchicine dose increase, paracetamol use, NSAID use, corticoteroid use, resting, ED admission, use of herbs, dietary changes, abdominal hot pack application, none, other choice if available.

## Statistical analysis

Data were analysed using the Statistical Package for the Social Sciences Statistics for Windows, version 26 (IBM Corp., Armonk, NY, USA). The normality of the variables was determined by analytical methods (Kolmogorov–Smirnov and Shapiro–Wilk). Non-parametric variables are expressed as median (interquartile range). Categorical variables are presented as numbers (percentage distributions). Comparative analyses were performed between the patients’ first choice attack mitigating strategies and the groups of education level, working status, colchicine response characteristics and adherence, ISSF, last attack duration, M694V positivity, CRP levels, and dominant attack types. The Pearson chi-square test or Fisher’s exact test (when the basic assumptions of the chi-square test are not met) was used to compare categorical variables between groups. For continuous variables that did not show a normal distribution, the Mann–Whitney U test was used in analytical comparisons. Type I error levels below 5% were considered statistically significant.

## Results

A total of 258 patients (98 males/160 females) were included in the study. The median age was 35 (19) years and the median disease duration was 18 (15) years. The median delay in diagnosis was 2 (9) years. The median age at symptom onset and age at diagnosis were 19 (16) and 13 (15) years, respectively. The most common educational level was 9–12 years (116 (45%)). 183 (70.9%) patients were working in any sector. A family history of FMF was present in 79.5% of patients. Clinical findings included peritonitis in 258 (100%) patients, pleuritis in 115 (44.6%), febrile myalgia in 77 (29.8%), fever in 232 (89.9%), erysipelas-like erythema in 100 (38.8%) and arthritis in 72 (27.9%). The dominant attack types of the patients were serositis in 118 (45%) patients and musculoskeletal attacks in 97 (37.5%) patients. Other clinical findings are shown in SI Table 1.

**Table 1:** First-line FMF attack mitigating strategies preferred by patients according to education level and working status

First-line strategies	≤ 8 years (n = 79)	9–12 years^2^ (n = 92)	> 12 years^3^ (n = 40)	p	Working (n = 183)	Non-working (n = 75)	p
Colchicine dose increase	12 (13.2)	20 (17.9)	15 (27.3)	0.101^a^	27 (14.8)	20 (26.7)	**0.024**^a^
Acetaminophen use	7 (7.7)	8 (7.1)	2 (3.6)	0.602^a^	8 (4.4)	9 (12)	**0.025**^a^
NSAIDs use	4 (4.4)	8 (7.1)	6 (10.9)	0.325^a^	9 (4.9)	9 (12)	**0.043**^a^
Corticosteroid use	6 (6.6)	6 (5.4)	0 (0)	0.155^a^	9 (4.9)	3 (4)	0.751^a^
Resting	10 (11)	19 (17)	8 (14.5)	0.482^a^	27 (14.8)	10 (13.9)	0.767^a^
ED admission	32 (35.2)^**2,3**^	13 (11.6)	6 (10.9)	** < 0.001**^a^	43 (23.5)	8 (10.7)	**0.019**^a^
Herbal agents*	6 (6.6)	8 (7.1)	3 (5.5)	0.918^a^	13 (7.1)	4 (5.3)	0.603^a^
Dietary changes**	4 (4.4)	4 (3.6)	3 (5.5)	0.922^a^	10 (5.5)	1 (1.3)	0.136^a^
Abdominal hot pack	3 (3.3)	8 (7.1)	3 (5.5)	0.558^a^	9 (4.9)	5 (6.7)	0.573^a^
On demand anakinra use	3 (3.3)	8 (7.1)	5 (9.1)	0.320^a^	16 (8.7)	0 (0)	**0.008**^a^
No factors	4 (4.4)	10 (8.9)	4 (7.3)	0.450^a^	12 (6.6)	6 (8)	0.680^a^

*FMF* familial Mediterranean fever, *NSAIDs* non-steroidal anti-inflammatory drugs, *ED* emergency department

^*^chamomile tea (n = 7), fennel tea (n = 6), lemon mint tea (n = 3)

^**^ restricting red meat (n = 6), restricting gluten (n = 5)

^2^Difference with the educational status 9–12 years group p < 0.05

^3^Difference with the educational status > 12 years group p < 0.05

Bold values indicate statistical significance (p < 0.05)

^a^Values given as n (%), Chi-Square test

A total of 252 (96.8%) patients had mutations in the MEFV gene. The most frequent mutation was M694V in 113 (51.6%) patients. M694V homozygous mutations were 50 (19.4%) in number and frequency. The rate of patients with the response to colchicine was 64.7%. The colchicine resistance was present in 27 (10.5%) patients. Thirty-five (13.6%) patients were receiving biologic therapy because of a history of colchicine resistance. The median duration of the last attack was 1 (1) day. The persistent CRP elevation level was present in 32.9% of patients (SI Table 1).

When the attack mitigation strategy groups were compared, it was found that the ISSF was significantly lower in the colchicine dose increase and no referral strategy groups (p < 0.001). Among ED admissions, ISSF was found to be significantly higher (p < 0.001). It was found that the last attack duration was significantly lower in the colchicine dose increase, NSAIDs use and no referral strategy groups. For ED admissions, the last attack duration was significantly longer (p < 0.05) (SI Table 2). In the ED admission group, the last attack duration ≥ 3 days (68.6%) and ISSF ≥ 4 (58.8%) were significantly higher (p < 0.001).

**Table 2:** First-line FMF attack mitigating strategies preferred by patients according to serositis and musculoskeletal attacks

First-line strategies	Serositis attacks group (n = 118)		p	Musculoskeletal attacks group (n = 97)		p
	No	Yes		No	Yes	
Colchicine dose increase	29 (20.7)	18 (15.3)	0.258^a^	36 (24.3)	11 (10.0)	**0.003**^a^
Acetaminophen use	13 (9.3)	4 (3.4)	0.057^a^	12 (8.1)	5 (4.5)	0.254^a^
NSAIDs use	11 (7.9)	7 (5.9)	0.545^a^	11 (7.4)	7 (6.4)	0.739^a^
Corticosteroid use	4 (2.9)	8 (6.8)	0.136^a^	6 (4.1)	6 (5.5)	0.597^a^
Resting	24 (17.1)	13 (11)	0.162^a^	24 (16.2)	13 (11.8)	0.319^a^
ED admission	21 (15)	30 (25.4)	**0.036**^a^	17 (11.5	34 (30.9)	** < 0.001**^a^
Herbal agents	10 (7.1)	7 (5.9)	0.696^a^	10 (6.8)	7(6.4)	0.900^a^
Dietary changes	6 (4.3)	5 (4.2)	0.985^a^	9 (6.1)	2 (1.8)	0.094^a^
Abdominal hot pack	5 (3.6)	9 (7.6)	0.152^a^	14 (9.5)	0 (0)	**0.001**^a^
On demand anakinra use	3 (2.1)	13 (11)	**0.003**^a^	3 (2)	13 (11.8)	**0.001**^a^
No factors	14 (10)	4 (3.4)	**0.038**^a^	6 (4.1)	12 (10.9)	**0.033**^a^

*FMF* familial Mediterranean fever, *NSAIDs* non-steroidal anti-inflammatory drugs, *ED* emergency department

Bold values indicate statistical significance (p < 0.05)

^a^Values given as n (%), Chi-Square test

**Fig. 1:**
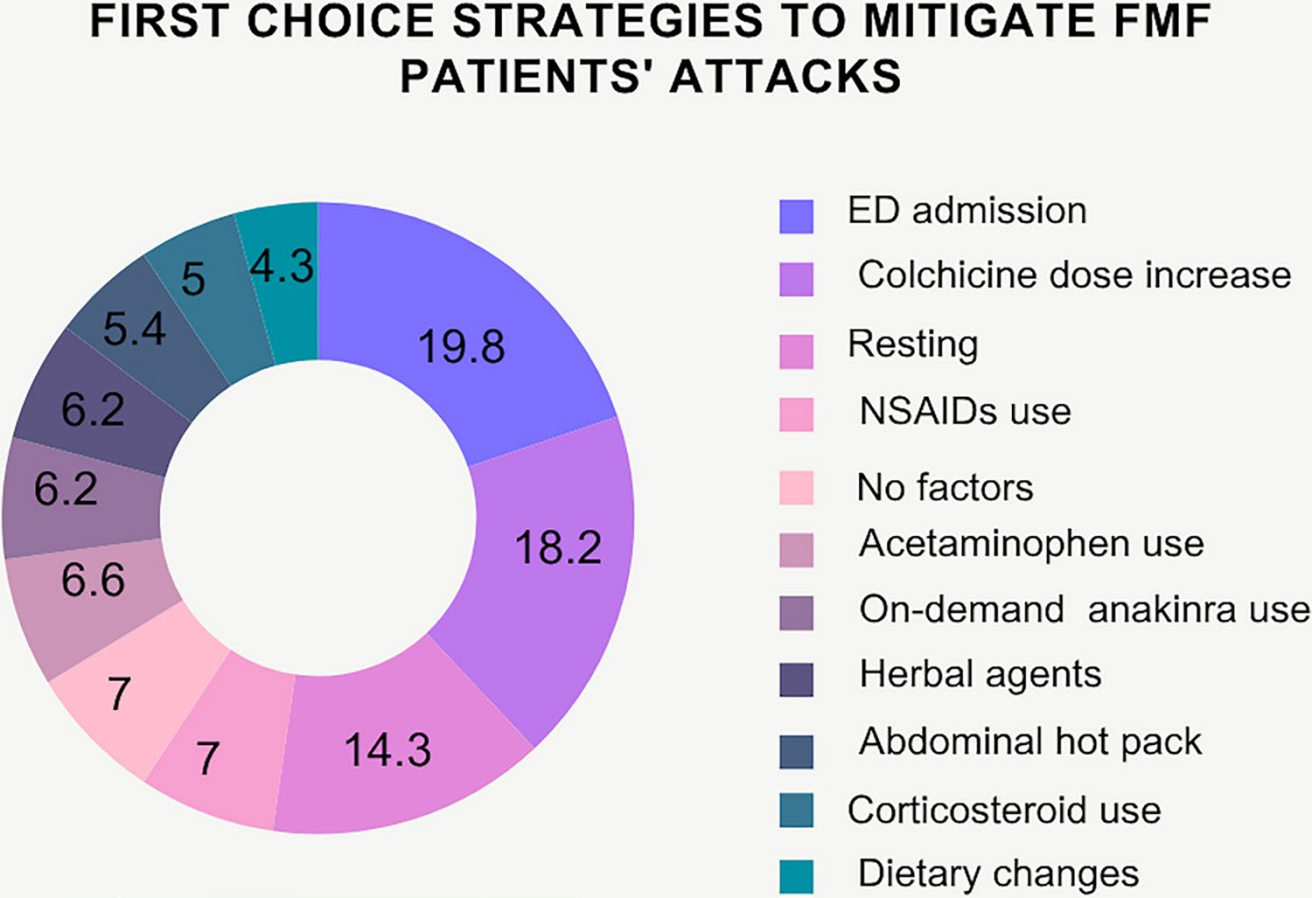
First choice strategies to mitigate FMF patients’ attacks. *FMF* familial Mediterranean fever, *ED* emergency department, *NSAIDs* non-steroidal anti-inflammatory drugs, Values given as %

A total of 240 patients (93%) reported a strategy to mitigate their attack. The most common first choice strategy was the ED admission (51 (19.8%)). Other strategies were resting in 37 (14.3%) patients, colchicine dose increase in 47 (18.2%) patients, paracetamol use in 17 (6.6%) patients, NSAIDs use in 18 (7%) patients, corticosteroid use in 13 (5%) patients, herbal tea use in 16 (6.2%) patients, dietary changes in 11 (4.3%) patients, abdominal hot packs in 14 (5.4%) patients, and on-demand use of anakinra in 16 (6.2%) patients. There was no referral strategy in 18 (7%) patients (see Fig. 1). The herbal agents used were chamomile (n = 7), fennel (n = 6) and lemon mint (n = 3) teas. Dietary changes included restriction of red meat in 6 patients and gluten in 5 patients.

When we compared the first-choice strategies that mitigate attacks according to education level, the rate of ED admissions was significantly higher among those with an education level of 8 years or less compared to other education levels (p < 0.001). When we compared the first-choice strategies that mitigate attacks according to working status, colchicine dose increase, acetaminophen and NSAIDs use were significantly higher in the non-working group (p = 0.024, 0.025 and 0.043, respectively). In the working group, on-demand use of anakinra and ED admission were significantly higher (p = 0.019, 0.008) **(**Table 1**).**

We compared the first choice strategies that mitigated attacks according to serositis and musculoskeletal attacks, and found that the rate of ED admissions and on-demand use of anakinra were significantly higher in those with serositis attacks (p = 0.036 and 0.003). In the group without serositis attacks, the rate of no referral strategy was significantly higher (p = 0.038). In the group with musculoskeletal attacks, the rates of ED admissions, no referral strategy and on-demand use of anakinra were significantly higher (p < 0.001, 0.001 and 0.033, respectively). In the group without musculoskeletal attacks, the rate of abdominal hot pack application was significantly higher (p = 0.001) **(**Table 2**).**

We also compared the first choice strategies according to M694V positivity and a persistent CRP elevation, the rate of ED admission was found to be significantly higher in the M694V positive group (p = 0.006). In the group without M694V positivity, colchicine dose increase and no application to any strategy were significantly higher (p < 0.001 and 0.002, respectively). The rate of ED admission was significantly higher in patients with a persistent CRP elevation (p < 0.001 and 0.041, respectively). Colchicine dose increase, resting and absence of preference strategy were significantly higher in patients without persistent CRP elevation (p < 0.001 and 0.010, respectively) **(**Table 3**)**.

**Table 3:** First-line FMF attack mitigating strategies preferred by patients with M694V positivity and persistent CRP elevation

First-line strategies	M694V positivity (n = 133)		p	Persistent CRP elevation (n = 63)		p
	No	Yes		No	Yes	
Colchicine dose increase	34 (27.2)	13 (9.8)	** < 0.001**^a^	61 (96.8)	2 (3.2)	** < 0.001**^a^
Acetaminophen use	8 (6.4)	9 (6.8)	0.905^a^	13 (6.7)	4 (6.3)	0.930^a^
NSAIDs use	5 (4)	13 (9.8)	0.069^a^	17 (8.7)	1 (1.6)	0.053^a^
Corticosteroid use	3 (2.4)	9 (6.8)	0.096^a^	8 (4.1)	4 (6.3)	0.462
Resting	19 (15.2)	18 (13.5)	0.703^a^	34 (17.4)	3 (4.8)	**0.013**^a^
ED admission	16 (12.8)	35 (26.3)	**0.006**^a^	13 (6.7)	38 (60.3)	** < 0.001**^a^
Herbal agents	8 (6.4)	9 (6.8)	0.905^a^	14 (7.2)	3 (4.8)	0.501^a^
Dietary changes	5 (4)	6 (4.5)	0.839^a^	7 (3.6)	4 (6.3)	0.346^a^
Abdominal hot pack	8 (6.4)	6 (4.5)	0.503^a^	12 (6.2)	2 (3.2)	0.364^a^
On demand anakinra use	4 (3.2)	12 (9)	0.053^a^	14 (7.2)	2 (3.2)	0.252^a^
No factors	15 (12)	3 (2.3)	**0.002**^a^	18 (9.2)	0 (0)	**0.012**^a^

*FMF* familial Mediterranean fever, *CRP* C-reactive protein, *NSAIDs* Non-steroidal anti-inflammatory drugs, *ED* Emergency department

Bold values indicate statistical significance (p < 0.05)

^a^Values given as n (%), Chi-Square test

Colchicine dose increase and no referral strategy were significantly higher in the colchicine complete response group compared to the other groups (p < 0.001 and 0.015, respectively). The rate of ED admission was significantly higher in the group with existing colchicine resistance and colchicine non-adherence (p < 0.001) **(**Table 4**).**

**Table 4:** First -line FMF attack mitigating strategies preferred by patients based on colchicine response and adherence

First-line strategies	Colchicine response^1^ (n = 167)	Current colchicine resistance^2^ (n = 27)	Colchicine non-adherent^3^ (n = 29)	Biologic therapy for colchicine resistance^4^ (n = 35)	p
Colchicine dose increase	47 (28.1)^**2,3,4**^	0 (0)	0 (0)	0 (0)	< **0.001**^a^
Acetaminophen use	13 (7.8)	0 (0)	3 (10.3)	1 (2.9)	0.348^b^
NSAIDs use	15 (9)	0 (0)	1 (3.4)	2 (5.7)	0.422^b^
Corticosteroid use	6 (3.6)	2 (7.4)	2 (6.9)	2 (5.7)	0.505^b^
Resting	26 (15.6)	2 (7.4)	1 (3.4)	8 (22.9)	0.110^a^
ED admission	12 (7.2)	20 (74.1)^**1,4**^	17 (58.6)^**1,4**^	2 (5.7)	** < 0.001**^a^
Herbal agents	12 (7.2)	0 (0)	2 (6.9)	3 (8.6)	0.544^b^
Dietary changes	7 (4.2)	2 (7.4)	2 (6.9)	0 (0)	0.334^b^
Abdominal hot pack	11 (6.6)	1 (3.7)	1 (3.4)	1 (2.9)	0.933^b^
On demand anakinra use	0 (0)	0 (0)	0 (0)	16 (45.7)^**1,2,3**^	** < 0.001**^b^
No factors	18 (10.8)^**2,3,4**^	0 (0)	0 (0)	0 (0)	**0.015**^b^

*FMF* familial Mediterranean fever, *NSAIDs* non-steroidal anti-inflammatory drugs, *ED* emergency department

^**1**^Difference with the colchicine response group p < 0.05

^**2**^Difference with the current colchicine resistance group p < 0.05

^**3**^Difference with the colchicine non-adherent group p < 0.05

^4^Difference with the biologic therapy group for colchicine resistance p < 0.05

Bold values indicate statistical significance (p < 0.05)

^a^Values given as n (%), Chi-Square test

^b^Values given as n (%), Fisher’s exact test

**Fig. 2:**
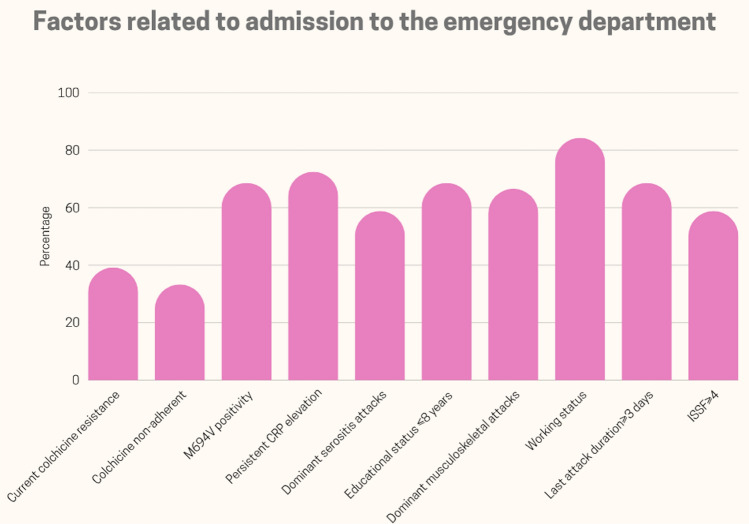
The variables linked to emergency department admission as a means of mitigating attacks in patients with FMF. *FMF* familial Mediterranean fever, *CRP* C-reactive protein, *ISSF* International Severity Score for FMF

In summary, the variables associated with ED admission were: M694V positivity, persistent CRP elevation, working status, serositis attacks, musculoskeletal attacks, an education level of eight years or less, a longer last attack duration, a higher ISSF, current colchicine resistance, and colchicine non-adherence (see Fig. 2).

## Discussion

To the best of our knowledge, this is the first study to evaluate the effectiveness of commonly used strategies for mitigating attacks in FMF patients, as well as the factors that influence the selection of these strategies. The high prevalence of patients using such strategies (93%) highlights the significant impact of FMF attacks on daily life and the need for effective management approaches. In the present study, the most common strategies used to mitigate attack severity were ED admission, followed by increasing colchicine dose, resting, NSAIDs use, paracetamol use, on-demand use of anakinra, herbal agents, corticosteroid use and dietary changes. Normal CRP levels, shorter last attack duration, lower ISSF, non-working and non-M694V mutations were associated with colchicine dose increase. M694V positivity, working, persistent CRP elevation, serositis and musculoskeletal attacks, longer last attack duration, higher ISSF, education level ≤ 8 years, current colchicine resistance and non-adherence were associated with ED admission.

While the standard dose of colchicine is effective for most patients, individual differences mean that adjustments must be made based on clinical response and side effects [1]. For most patients, the optimal daily dose required to prevent AA amyloidosis and attacks is 1–1.5 mg. However, if an adequate response is not achieved, the dose may be increased to a maximum of 3 mg per day [25]. However, despite receiving the maximum tolerated dose of colchicine, 5–10% of patients do not respond well to treatment [4]. The plasma colchicine levels of FMF patients are influenced by various factors, including dosage, genetics, body surface area, adherence to treatment, and pharmacokinetic properties [26]. A study evaluating treatment strategies for FMF attacks related to menstruation found that increasing the colchicine dose and switching from coated to compressed tablets were the most common strategies employed [27]. The EULAR treatment recommendations suggest increasing the dose of colchicine for FMF attacks triggered by emotional or physical stress [25]. In this study, 93% of FMF patients reported using strategies to alleviate attacks. The most plausible reason for resorting to these strategies appears to be an inability to achieve the optimal colchicine dose, given that most patients without colchicine resistance or non-compliance reported increasing their colchicine dose during attacks to manage them. However, to establish this context clearly, plasma colchicine levels need to be measured, though this is not routinely done in practice. Canbolat and colleagues reported that an optimal clinical response was observed at colchicine doses of 1.5–2 mg/day, corresponding to plasma levels of approximately 1.097 ± 0.42 ng/ml [27]. Other causes that we identified include colchicine resistance and non-compliance with treatment. Patients experiencing these issues often require ED admissions or biological treatment due to more frequent and severe pain attacks. The most commonly recommended method of improving colchicine compliance is administering a low dose daily. Additionally, when non-compliance with colchicine is present, monitoring blood drug levels and investigating potential drug interactions is recommended [28, 29]. Additionally, this study found that attack duration and ISSF scores were lower in the group that increased their colchicine dosage. This finding supports the treatment response. However, it emphasises the need to consider reasons for incomplete responses, as the attack is still present. While it was not possible to measure the drug level in this study, other parameters were evaluated and a drug adherence improvement plan was developed.

In this study, ED admission emerged as the most common first choice. ED admissions play an important role in the initial diagnosis and treatment of FMF, particularly in areas where the disease is prevalent. While ED can provide immediate relief for symptoms such as pain and fever, they cannot treat FMF itself. The primary treatment for FMF involves taking medications such as colchicine long term to help prevent attacks and complications [14]. Masatlioglu et al. [14] reported that 2% of patients presenting with acute abdominal pain were diagnosed with FMF. They noted that this rate was significantly higher than that observed in the general Turkish population. A study of 1,788 individuals presenting to ED with a rheumatic disease diagnosis found that FMF was the most common condition after ankylosing spondylitis and rheumatoid arthritis, accounting for 18.1% of cases. FMF symptoms were the reason for presentation to ED in 88.5% of patients with FMF. The rate of referral due to rheumatological symptoms was lower than in other diseases [30].

Patients with a low level of education are more likely to use ED [31]. People with low levels of education tend to experience poorer health outcomes, which can lead to an increase in ED admission [32]. In this study, patients with lower educational levels also visited ED more frequently.

Sleep disorders and daytime fatigue are directly related to work stress factors. These factors include working hours, high job demands and low job control [33]. Fatigue and sleep disorders have also been reported to trigger FMF attacks [34]. This study found that working in any occupation was associated with ED admission. This could be because emergency care provides rapid symptom control, thereby increasing work productivity, or because work triggers attacks in relation to the aforementioned factors.

The M694V mutation, particularly in its homozygous form, has been associated with an earlier onset of symptoms, a greater frequency of attacks and a more severe progression of the disease. [4]. These reasons may explain why patients prefer ED admission for attack management. In this study, M694V positivity was also found to be associated with ED admission. In line with all these associations, it is expected that the last attack duration and ISSF would be higher in the ED admission group.

The on-demand use of anakinra has been investigated in the literature, with its benefits noted in the treatment of colchicine-resistant FMF patients. This approach is particularly useful for patients with distinct attack triggers or prodromal symptoms, as it allows for targeted intervention [9]. In this study, on-demand use of anakinra was associated with dominant serositis and musculoskeletal attacks, reflecting its role as a rescue therapy for severe inflammatory attacks. In this study, triggering factors were present in the majority of patients who used anakinra on demand.

Non-opioid analgesics, such as NSAIDs and paracetamol, are often used in ED to treat acute pain, as they are less potent and have fewer side effects than opioids. [35]. The use of NSAIDs during attacks is recommended by EULAR treatment guidelines [25]. A study evaluating treatment strategies for FMF attacks related to menstruation found that the methods of treatment involving steroids, NSAIDs and anakinra were effective in preventing attacks or reducing their severity [36]. In the study by Akar and et al. [37], NSAIDs were not effective in premenstrual attacks. Giese et al. [12] reported that NSAIDs and paracetamol were the most effective drugs among those used to relieve pain during attacks in 80 patients with FMF, including opioid analgesics. In a non-randomised, single-blind, placebo-controlled study, it was found that moderate-dose intravenous steroid treatment initiated within the first 24 h of an acute attack was effective in controlling the attack [10]. Additionally, the shorter duration of the last attack in the NSAID group is likely due to NSAIDs suppressing acute inflammation.

The use of NSAIDs, paracetamol and corticosteroids in this study suggests that pain management is an important aspect of attack relief.

Other strategies such as resting, dietary changes, abdominal hot pack and herbs use were less preferred in this study. Hot packs are one of the most common methods of pain relief [38]. To the best of our knowledge, its use in treating FMF has not been documented in the literature.

The use of complementary therapies among people with rheumatic diseases has been found to range from 22 to 95%. In Türkiye, the most commonly used methods are nutrition, herbal remedies, mineral and vitamin supplements, religious practices such as prayer and cupping, and hydrotherapy [39]. A study investigating the relationship between the use of alternative and integrative treatments and symptoms in Turkish FMF patients found that 24.6% of patients reported taking herbal supplements, including turmeric, ginger, rosemary and green tea [39]. However, there is insufficient data on the use of herbs to mitigate the severity only during the attack period. In this study, albeit at a low rate, patients used herbal teas such as chamomile, fennel and mint lemon to reduce the severity of attacks. Anti-inflammatory, analgesic, anxiolytic and sedative effects of chamomile are known [40, 41]. Emotional stress is a known trigger for FMF attacks [42]. The anxiolytic properties of chamomile may therefore help to manage stress levels, potentially reducing the frequency or severity of attacks. Fennel and mint lemon have been shown to have gastrointestinal, anti-inflammatory and analgesic effects [43–45]. In conclusion, it can be said that the patients who participated in this study used the herbals in question for their anxiolytic, analgesic and anti-inflammatory properties.

The relationship between diet and FMF is complex. Literature suggests that wheat, salty and fatty foods may play a role in FMF attacks, but this is inconclusive. Anti-inflammatory supplements and a diet rich in antioxidants may help reduce symptoms and improve well-being, but evidence is limited [16]. The question of whether dietary changes, particularly a reduction in red meat and gluten, can reduce the severity of FMF attacks is complicated by the fact that current research is primarily focused on other inflammatory conditions. These studies suggest that dietary changes, such as reducing red meat and gluten intake, may affect inflammation and symptom severity in some cases [46, 47]. The role of diet in FMF still needs to be proven in randomised controlled trials.

Interestingly, some patients did not opt for any intervention. This was associated with non-M694V mutations, normal CRP levels and fewer episodes of serositis. This suggests that milder disease may require less aggressive treatment. Furthermore, the shorter last attack duration and lower ISSF score of this group lend weight to this view.

### Limitations

Due to the nature of cross-sectional studies, the ability to establish causality between mitigation strategies and patient outcomes is limited. Conducting the study in a single tertiary rheumatology clinic may limit the generalisability of the results to broader FMF populations, especially populations in different geographical settings. Relying on patients’ recollection of their strategies over the past six months could introduce ‘recall bias’. Additionally, the modified Morisky scale is a subjective method of assessing medication adherence, which could be considered a limitation. More objective measures, such as colchicine blood levels, could offer additional insights into adherence and pharmacokinetics. Another limitation is the failure to record residence information, which could prevent patients from accessing health services. These limitations should be considered when interpreting the results, and future studies can address these limitations by combining multicentre data and prospective designs.

## Conclusion

These findings suggest that personalised treatment plans that take into account genetic, sociodemographic, inflammatory and educational strategies may improve FMF management. Increasing patient education, improving colchicine adherence and optimising the use of biologic agents can help reduce ED admissions and improve disease control. These approaches can improve outcomes for patients with FMF by providing more effective and patient-centred care.

## Supplementary Information

Below is the link to the electronic supplementary material.

Supplementary file1 (DOCX 25 KB)

## Data Availability

The datasets utilized and/or analyzed in this investigation are obtainable from the corresponding author upon reasonable request.
