# Supplementary material for: What do patients do during a familial Mediterranean fever attack? Their strategies and associated factors
Source: Intern Emerg Med. 2025 Jul 1;20(6):1823–33. doi: 10.1007/s11739-025-04039-6 (PMC12476397; doi:10.1007/s11739-025-04039-6)
Supplement: Supplementary file 1 — Supplementary file1 (DOCX 25 KB) [file 11739_2025_4039_MOESM1_ESM.docx]

**S table 1. The clinical characteristics of FMF patients**

| Parameters | Patients  (n=258) |
| --- | --- |
| Age, years, median (IQR) | 35 (19) |
| Gender, female/male, n (%) | 160 (62)/98 (38) |
| Age at symptom onset, years, median (IQR) | 19 (16) |
| Age at diagnosis, years, median (IQR) | 13 (15) |
| **Diagnosis** duration, years, median (IQR) | 13 (13) |
| **Disease duration,**  years, median (IQR) | 18 (15) |
| Time from symptom onset to diagnosis, median (IQR) | 2 (9) |
| Educational Status, n (%) |  |
| ≤8 years | 8 (34.5) |
| 9–12 years | 116 (45) |
| >12 years | 53 (20.5) |
| Working status, n (%) | 183 (70.9) |
| Family history of FMF, n (%) | 205 (79.5) |
| History of appendectomy, n (%) | 60 (23.3) |
| Parental consanguinity, n (%) | 77 (29.8) |
| Clinical findings, n (%) |  |
| Peritonitis | 242 (93.8) |
| Pleuritis | 115 (44.6) |
| Pericarditis | 27 (10.5) |
| Fever | 232 (89.9) |
| Arthritis | 72 (27.9) |
| Erysipelas-like erythema | 100 (38.8) |
| Febrile myalgia | 77 (29.8) |
| Diarrhea | 76 (29.5) |
| Colchicine dose, mg/day, median (IQR) | 1.5 (1) |
| Dominant attack type, n (%) |  |
| Serositis | 118 (45.7) |
| Musculoskeletal | 97 (37.5) |
| Current colchicine resistant patients, n (%) | 27 (10.5) |
| Colchicine non-adherent patients, n (%) | 29 (11.2) |
| Colchicine responsive patients, n (%) | 167 (64.7) |
| Biologic therapy for colchicine resistance, n (%) | 35 (13.6) |
| Last attack duration, days, median (IQR) | 1 (1) |
| Disease severity (ISSF score), median (IQR) | 2 (3) |
| Persistent CRP elevation | 63 (24.4) |
| Normal CRP level | 195 (75.6) |
| MEFV gene mutations, n (%) |  |
| Exon 10/Exon10 | 89 (34.5) |
| Exon 10/- | 119 (46.1) |
| Exon 10/Exon 2 | 12 (4.7) |
| Exon 2/ Exon 2 | 1 (0.4) |
| Exon 2/ - | 33 (12.8) |
| Homozygous mutation | 59 (22.9) |
| M694V homozygosity | 50 (19.4) |
| M694V positivity | 133 (51.6) |
| No mutation | 6 (2.3) |

**Abreviations**: IQR: Interquartile range; FMF: Familial Mediterranean fever; ISSF: International Severity Score for FMF, CRP: C-reactive protein.

| **First -line strategies** | **ISSF** | | **p** | **Last attack duration*** | | **p** |
| --- | --- | --- | --- | --- | --- | --- |
| Colchicine dose increase, yes/no^a^ | 0 (1) | 2 (3) | <**0.001**^a^ | 1 (0) | 1 (2) | <**0.001**^a^ |
| Acetaminophen use, yes/no^a^ | 2 (3) | 2 (3) | 0.887^a^ | 1 (1) | 1 (1) | 0.810^a^ |
| NSAIDs use, yes/no^a^ | 1.5 (2) | 2 (3) | 0.345^a^ | 1 (0) | 1 (1) | **0.022**^a^ |
| Corticosteroid use, yes/no^a^ | 2.5 (4) | 1 (3) | 0.220^a^ | 1.5 (1.7) | 1 (1) | 0.415^a^ |
| Resting, yes/no^a^ | 1 (2) | 2 (3) | 0.097^a^ | 1 (1) | 1 (2) | 0.214^a^ |
| ED admission, yes/no^a^ | 4 (3) | 1 (2) | <**0.001**^a^ | 3 (2) | 1 (1) | <**0.001**^a^ |
| Herbal agents, yes/no^a^ | 2 (2) | 1 (3) | 0.528^a^ | 1 (1) | 1 (1) | 0.531^a^ |
| Dietary changes, yes/no^a^ | 1 (4) | 2 (3) | 0.847^a^ | 2 (3) | 1 (1) | 0.160^a^ |
| Abdominal hot pack, yes/no^a^ | 2 (2.2) | 1.5 (3) | 0.921^a^ | 1 (1.2) | 1 (1) | 0.794^a^ |
| On demand anakinra use, yes/no^a^ | 2 (1) | 1 (3) | 0.020^a^ | 1 (1.2) | 1 (1) | 0.619^a^ |
| No factors, yes/no^a^ | 0 (0.2) | 2 (3) | <**0.001**^a^ | 1 (0) | 1 (1.7) | **0.002**^a^ |

**S table 2.** Comparison of the International Severity Score for FMF and the duration of the last

attack between attack mitigation strategy groups

**Abreviations**: FMF: Familial Mediterranean fever; ISSF: International Severity Score for FMF; NSAIDs: Non-steroidal anti-inflammatory drugs; ED: Emergency department; ***** days; Bold values indicate statistical significance (p < 0.05) . ^a^Values given as median (interquartile range), Mann-Whitney U test
